# Strategies Shaping the Transcription of Carbohydrate-Active Enzyme Genes in *Aspergillus nidulans*

**DOI:** 10.3390/jof8010079

**Published:** 2022-01-14

**Authors:** Barnabás Cs. Gila, Károly Antal, Zsuzsanna Birkó, Judit Sz. Keserű, István Pócsi, Tamás Emri

**Affiliations:** 1 Department of Molecular Biotechnology and Microbiology, Faculty of Sciences and Technology, University of Debrecen, Egyetem tér 1, 4032 Debrecen, Hungary; gila.barnabas@science.unideb.hu (B.C.G.); pocsi.istvan@science.unideb.hu (I.P.); 2 Doctoral School of Nutrition and Food Sciences, University of Debrecen, Egyetem tér 1, 4032 Debrecen, Hungary; 3 Department of Zoology, Eszterházy Károly Catholic University, Eszterházy tér 1, 3300 Eger, Hungary; antalk2@gmail.com; 4 Department of Human Genetics, Faculty of Medicine, University of Debrecen, Egyetem tér 1, 4032 Debrecen, Hungary; birko@med.unideb.hu (Z.B.); keseru.judit@med.unideb.hu (J.S.K.)

**Keywords:** arabinogalactan, *Aspergillus nidulans*, carbohydrate-active enzyme, carbon limitation, carbon starvation, sterigmatocystin production, transcriptomics, utilization of lactose

## Abstract

Understanding the coordinated regulation of the hundreds of carbohydrate-active enzyme (CAZyme) genes occurring in the genomes of fungi has great practical importance. We recorded genome-wide transcriptional changes of *Aspergillus nidulans* cultivated on glucose, lactose, or arabinogalactan, as well as under carbon-starved conditions. We determined both carbon-stress-specific changes (weak or no carbon source vs. glucose) and carbon-source-specific changes (one type of culture vs. all other cultures). Many CAZyme genes showed carbon-stress-specific and/or carbon-source-specific upregulation on arabinogalactan (138 and 62 genes, respectively). Besides galactosidase and arabinan-degrading enzyme genes, enrichment of cellulolytic, pectinolytic, mannan, and xylan-degrading enzyme genes was observed. Fewer upregulated genes, 81 and 107 carbon stress specific, and 6 and 16 carbon source specific, were found on lactose and in carbon-starved cultures, respectively. They were enriched only in galactosidase and xylosidase genes on lactose and rhamnogalacturonanase genes in both cultures. Some CAZyme genes (29 genes) showed carbon-source-specific upregulation on glucose, and they were enriched in β-1,4-glucanase genes. The behavioral ecological background of these characteristics was evaluated to comprehensively organize our knowledge on CAZyme production, which can lead to developing new strategies to produce enzymes for plant cell wall saccharification.

## 1. Introduction

Fungi can use a wide range of organic compounds as a carbon and energy source. The spatial and temporal availability of these compounds is highly variable in most natural habitats where fungi occur. Not surprisingly, carbon stresses (carbon-starvation stress and carbon-limitation stress) are among the stresses that most affect the life of fungi [1,2,3]. The type and availability of carbon/energy sources highly influence many aspects of fungal life, including the production of carbohydrate-active enzymes (CAZymes), secretion of extracellular hydrolases (other than CAZymes) [2,4], formation of secondary metabolites (including mycotoxins or molecules with pharmaceutical interest) [5,6], as well as their sexual and asexual differentiation, stress tolerance, and even their antifungal susceptibility and pathogenicity [7,8,9,10]. Understanding carbon-starvation stress responses, hence, has great practical importance both in the fermentation industry and in medical mycology.

The plant cell wall consists of several polysaccharides including cellulose (and in smaller portions, mixed-linkage glucan), hemicelluloses (e.g., xylans, xyloglucans, mannans), and pectins [11]. In addition to these, plant cell walls contain different hydroxyproline-rich peptides, such as extensins and arabinogalactan proteins, harboring a substantial portion of saccharide residues [11]. Many fungi are able to utilize these polysaccharides. They secrete extracellular enzymes to cut (usually hydrolyze) these polymers into parts (usually oligomers/monomers), and these smaller molecules are taken up for further degradation/utilization. Efficient degradation of complex saccharides generally needs multiple, synergistically acting enzymes [4,12,13]. Extracellular degradation of polysaccharides lets cells produce the needed amount of enzymes in cooperation. Moreover, the mono- and oligosaccharides liberated extracellularly function as “public goods,” since they can be utilized by any microbes in the vicinity, even by those called “cheaters,” who have not secreted any enzymes contributing to the degradation. Not surprisingly, extracellular enzyme production of microbes is intensively studied by behavioral ecologists [14]. Efficient saccharification of plant polysaccharides is crucial in the cost-efficient production of second-generation biofuels [15]. Fungi are important sources of various industrially important enzymes including those that can be used for saccharification of complex plant materials. Understanding how fungi adapt to different carbon/energy sources can help to identify new genes or molecules involved in the regulation of enzyme production as well as new (auxiliary) enzymes for efficient degradation of these biopolymers. This knowledge can be used to improve the efficiency of plant cell wall saccharification or to reduce the production cost of the enzymes used [15,16,17].

*Aspergillus nidulans,* like many other filamentous fungi, commonly occur in habitats rich in decaying plant materials. Its genome contains more than 400 CAZyme genes (Carbohydrate-active Enzymes Database; http://www.cazy.org/; 10 December 2021). Thanks to these, it can grow efficiently on complex plant materials [18,19,20]. Not surprisingly, it is not only a model organism but also a potential source of various enzymes for industrial application [21].

Here, we studied genome-wide transcriptional changes in four *A. nidulans* cultures differing in the available carbon/energy source: cultures incubated in glucose-rich or carbon-source-free media, as well as cultures growing on lactose or arabinogalactan. We aimed to explain the transcriptional changes of CAZyme genes as well as the physiology of the fungus using the following four strategies connected to the formation and utilization of public goods: (1) “secretion of scouting enzymes” [22,23,24]; (2) “adaptive prediction” in stress responses [25,26]; (3) prevention of the rise of “cheaters” [14]; and (4) Garrett Hardin’s “tragedy of the commons” scenario [14].

## 2. Materials and Methods

### 2.1. Strains and Culture Conditions

The *A. nidulans* THS30 (*pyrG89*; *AfupyrG^+^*; *pyroA4*; *pyroA^+^*) reference strain [27] was used in this study. It was maintained on Barratt’s minimal agar plates at 37 °C [28] and only freshly collected conidia from 6 d cultures were used for inoculation in all experiments. For submerged cultivation, 500 mL Erlenmeyer flasks containing 100 mL Barratt’s minimal broth were inoculated with 50 × 10^6^ conidia/flask and were incubated at 37 °C and 3.7 Hz (approx. 220 rpm) shaking frequency for 16 h. The exponentially growing phase mycelia were collected by filtration, washed, and then transferred into 100 mL fresh Barratt’s minimal broth. These media contained 20 g L^−1^ glucose, 20 g L^−1^ lactose, or 20 g L^−1^ arabinogalactan (from larch wood, Sigma-Aldrich Ltd., Budapest, Hungary) as carbon/energy source, or did not contain any carbon source at all. All cultures were further incubated at 37 °C and 3.7 Hz shaking frequency.

### 2.2. Detecting Growth, Metabolic Activity, Carbohydrate Utilization, and Formation of Sterigmatocystin

Growth of the cultures was monitored by measuring their dry cell mass (DCM) content [29]. The metabolic activity of the hyphae was characterized by their methylthiazoletetrazolium (MTT) reduction activity as described previously [29]. Glucose, lactose, and arabinogalactan consumption was followed by detecting the reducing sugar content of the media with *p*-amino-hydroxybenzoic acid hydrazide [30], while sterigmatocystin production was demonstrated by thin-layer chromatography (TLC) [31].

### 2.3. Enzyme Assays

Catalase and superoxide dismutase (SOD) activities were measured by rate assays [32,33] either with cell-free extracts prepared by X-pressing [34] or with the fermentation broth. Intracellular glutathione reductase, nitrate reductase, and β-galactosidase activities were determined according to Pinto et al. [35], Bruinenberg et al. [36], and Nagy et al. [37], respectively, from the cell-free extracts. Extracellular chitinase [34], N-acetyl-glucosaminidase [38], β-glucosidase [30], cellulase [30], γ-glutamyl transpeptidase (γGT) [39], and protease [40] activities were determined from the fermentation broth. In the case of cellulase determination, which was based on measuring the increase in the reducing sugar content in the carboxymethyl-cellulose substrate solution, fermentation broth was dialyzed against 0.1 mol L^−1^ K-phosphate buffer (pH 6.5) prior to measurement to remove reducing sugars. Protein content of the cell-free extracts was measured with Bradford reagent. In the case of the glucose-containing cultures, all enzyme activities were determined at 4 h to prevent biases caused by the quick decrease in the glucose content of the cultures. In the case of the carbon-stressed cultures, specific SOD, catalase, nitrate reductase, glutathione reductase, and β-galactosidase activities were measured at 12 h. All the other (extracellular) enzyme activities were determined using 24 h cultures, since in the case of these enzymes the activity values were too low in the 12 h cultures for precise determination.

### 2.4. Reverse-Transcription Quantitative Real-Time Polymerase Chain Reaction (RT-qPCR) Assays

Lyophilized mycelia were used to isolate total RNA according to Chomczynski [41]. RT-qPCR assays were carried out with the primer pairs listed in Table S1 using Luna^®^ Universal One-Step RT-qPCR Kit (New England Biolabs, Ipswich, MA, USA) following the manufacturer’s protocol. Relative transcription levels were characterized with the ΔCP (difference between the crossing point of the reference and target gene within a sample) values using the AN6700 gene (putative translation elongation factor) as reference [42].

### 2.5. High throughput RNA Sequencing

Total RNA was isolated from lyophilized mycelia [41] from four different cultures using three biological replicates:

For “fast-growing” cultures, exponentially growing phase mycelia were transferred into Barratt’s minimal broth containing 20 g L^−1^ glucose as carbon/energy source and were incubated for 4 h at 37 °C and 3.7 Hz shaking frequency.

For “carbon-starved” cultures, exponentially growing phase mycelia were transferred into carbon-source-free Barratt’s minimal broth and were incubated for 12 h at 37 °C and a 3.7 Hz shaking frequency.

For “carbon-limited” cultures, exponentially growing phase mycelia were transferred into Barratt’s minimal broth containing either 20 g L^−1^ lactose or 20 g L^−1^ arabinogalactan and were incubated for 12 h at 37 °C and 3.7 Hz shaking frequency.

RNA sequencing (from library preparation to generation of fastq.gz files) as well as the RT-qPCR assays was carried out at the Genomic Medicine and Bioinformatic Core Facility, Department of Biochemistry and Molecular Biology, Faculty of Medicine, University of Debrecen, Debrecen, Hungary. A single-read 75 bp Illumina sequencing was performed as described previously [43]. All library pools were sequenced in the same lane of a sequencing flow cell, and 11.5–20.8 million reads per sample were obtained. The FastQC package (http://www.bioinformatics.babraham.ac.uk/projects/fastqc; 10 December 2021) was used for quality control. The hisat2 software (version 2.1.0) was used to align reads to the genome of *A. nidulans* FGSC A4 (genome: http://www.aspergillusgenome.org/download/sequence/A_nidulans_FGSC_A4/archive/A_nidulans_FGSC_A4_version_s10-m04-r12_chromosomes.fasta.gz; 1 September 2021; genome features file (GFF): http://www.aspergillusgenome.org/download/gff/A_nidulans_FGSC_A4/archive/A_nidulans_FGSC_A4_version_s10-m04-r12_features_with_chromosome_sequences.gff.gz; 1 September 2021;). More than 93% of reads were successfully aligned in the case of each sample. Differentially expressed genes were determined with DESeq2 (version 1.24.0).

### 2.6. Evaluation of the Transcriptome Data

When the transcriptomes of two cultures were compared (“A” vs. “B”), upregulated and downregulated genes were defined as genes that had shown significantly different expression (adjusted *p*-value < 0.05) and log_2_FC > 1 or log_2_FC < −1, respectively, where FC (fold change) stands for the number calculated by the DESeq2 software using “B” as the reference culture. In the case of carbon-starved cultures as well as cultures growing on arabinogalactan or lactose, “carbon-stress-responsive” genes were regarded as upregulated or downregulated genes coming from comparisons where glucose-containing cultures were used as reference. Genes were regarded as “culture specifically” upregulated (or downregulated) if they were upregulated (or downregulated) relative to the other three cultures.

The composition of carbon-stress-responsive and culture-specific gene sets was studied with gene set enrichment analyses carried out with “Functional Catalogue” (FunCat), “Gene Ontology” (GO), and “Kyoto Encyclopedia of Genes and Genomes pathway” (KEGG pathway) terms using the FungiFun2 package (https://elbe.hki-jena.de/fungifun/fungifun.php; 10 December 2021), applying default settings. Hits with a corrected *p*-value < 0.05 were regarded as significantly enriched and were taken into consideration during evaluation. Terms containing less than three genes or hits with only one gene were omitted from the analysis.

The enrichment of genes belonging to the groups defined below was tested by the Fisher’s exact test with the “fisher.test” function of R project (www.R-project.org/; 10 December 2021). The following gene sets were studied (the composition of the gene sets is available in Tables S2 and S3):

“Lactose utilization” genes: This gene group contains genes involved in the Leloir and oxido-reductive pathways of galactose utilization, the *galX* (AN10543) and *galR* (AN7610) genes encoding the transcriptional regulators of the above-mentioned pathways [44], as well as known and putative β-galactosidase and lactose permease genes according to Fekete et al. [45,46] and to the *Aspergillus* Genome database (AspGD; www.aspergillusgd.org; 1 September 2021).

“Autophagy” genes: Genes collected from AspGD with “autophagy protein” or “autophagy-related protein” terms.

“Antioxidant enzyme” genes: Genes of known or putative superoxide dismutases, catalases, peroxidases, or the glutathione/glutaredoxin/thioredoxin redox system collected from the AspGD.

“Squalene—ergosterol” pathway genes: Orthologues of *A. fumigatus* genes encoding enzymes of the squalene—ergosterol pathway [47] collected from AspGD.

“Glycolysis” genes, “Oxidative pentose-phosphate shunt” genes, “Ribose metabolism” genes, and “TCA cycle” genes: Genes described by Flipphi et al. [48].

“Extracellular peptidase” genes: Peptidase genes encoding N-terminal signal sequence but not transmembrane domain collected from the AspGD.

“Cell wall” genes: Genes of chitin synthases, glucan synthases, transglucosylases, endo-mannanases, chitinases, chitin deacetylases, hexosaminidases, glucanases, glucosidases, as well as genes of the cell wall integrity pathway and genes necessary for UDP-N-acetylglucosamine synthesis collected by de Groot et al. [49] and supplemented with N-acetyl-glucosamine transmembrane transporter (AN1427), N-acetyl-glucosamine-6-phosphate deacetylase (AN1428), and glucosamine-6-phosphate deaminase (AN1418) genes (genes necessary for N-acetylglucosamine utilization).

“Carbohydrate-active enzyme” (CAZyme) genes: Genes collected from the Carbohydrate-active Enzymes Database (http://www.cazy.org/; 10 December 2021), except the genes presented in the “Cell wall” genes group were omitted.

“Ribosome biogenesis” genes, “Mitotic cell cycle” genes, and “Transcription factor” genes: These groups were constructed based on the AspGD using the related GO terms and their child terms.

“Secondary metabolism cluster” genes: Manually or experimentally determined secondary metabolite cluster genes collected by Inglis et al. [50]. Gene set enrichment analysis was carried out with the clusters separately. Clusters where the upregulated or downregulated genes were significantly enriched (Fisher’s exact test, *p* < 0.05) in the appropriate gene set were regarded as upregulated or downregulated clusters, respectively.

### 2.7. Identification of Extracellular Proteins

Proteins from the fermentation broth were separated by 2D gel electrophoresis [51]. Selected spots were cut from gels manually, and, after trypsin digestion, peptides were analyzed on a 4000 QTRAP (AB Sciex, Framingham, MA, USA) mass spectrometer coupled to an Easy nLC II nanoHPLC (Bruker, Billerica, MA, USA). The obtained LC–MS/MS data were used for protein identification based on the ProteinPilot 4.5 (ABSciex) search engine, and the SwissProt database and minimum two peptides with 99% confidence were required for identification of the protein [39].

## 3. Results

### 3.1. Three Carbon Stress Types Caused Similar Physiological Changes

**Figure 1:**
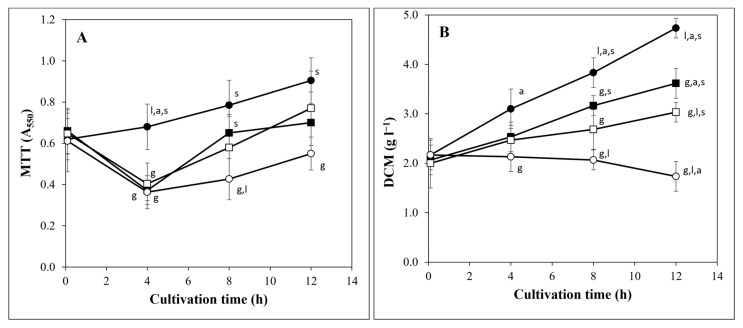
Growing and metabolic activity of the studied *A. nidulans* THS30 cultures. Changes in the MTT-reducing activity (**A**) and DCM (**B**) of the strain were characterized in cultures growing on glucose (●), in the absence of any carbon source (○), on lactose (■), or on arabinogalactan (□). Mean ± SD values calculated from three biological replicates are presented. The MTT-reducing activity of 12 h cultures was significantly higher (Student’s *t*-test, *p* < 0.05) than that of the 4 h cultures. The DCM of the 12 h glucose, lactose, or arabinogalactan-containing cultures was significantly higher (Student’s *t*-test, *p* < 0.05) than that of the 0 h cultures. ^g, l, a, s^—Significant difference (Student’s *t*-test, *p* < 0.05) from the data of cultures containing glucose, lactose, arabinogalactan, or no carbon source, respectively.

Physiological consequences of three different types of carbon stress (carbon-starvation stress as well as carbon-limitation stresses in the presence of either a disaccharide or a polysaccharide) were studied. Transferring mycelia from glucose-containing media to carbon-source-free or lactose/arabinogalactan-containing media significantly decreased the MTT-reducing activity of the cultures (Figure 1A). However, this decrease was temporary; after 4 h, MTT-reducing activity of the carbon-stressed cultures started to increase even in the case of the carbon-starved cultures (Figure 1A). Carbon stress also reduced the growth of the fungus as expected (Figure 1B). The highest reduction in biomass production, with respect to glucose-containing cultures, was observed in carbon-starved cultures followed by arabinogalactan and lactose-containing cultures. Although long-term carbon starvation is generally accompanied with DCM decline [34], only a small (but not statistically significant) decrease in the DCM was observed at 12 h in this case (Figure 1B).

Carbon stress significantly decreased the nitrate reductase and glutathione reductase activities in all cultures, while the intracellular β-galactosidase activities were elevated by carbon stress (Table 1). This increase was the strongest on lactose followed by arabinogalactan-containing and carbon-starved cultures (Table 1).

The intracellular SOD activities were increased on arabinogalactan and in carbon-starved cultures. The fermentation broth of all the four cultures had detectable SOD activity and the carbon-stressed cultures had detectable catalase activity as well (Table 1), which concur with the results of Saykhedkar et al. [18] who studied the secretome of *A. nidulans* cultivated on sorghum stover. Carbon stress increased extracellular proteinase, γGT, chitinase, and β-glucosidase activities (Table 1). Growing on lactose or on arabinogalactan but not carbon starvation also increased extracellular β-galactosidase and cellulase activities (Table 1).

In order to identify further enzymes from the fermentation broth of carbon-starved cultures, extracellular proteins were separated by 2D gel electrophoresis and the protein content of selected patches was analyzed. The presence of the following proteins in the fermentation broth was demonstrated (Table S4): AbnC (AN8007, putative extracellular endo-1,5-α-L-arabinosidase); EglB (AN3418, cellulase); BglA and BglL (AN4102 and AN2828; putative β-glucosidases); ChiB (AN4871, chitinase); EglC (AN7950, putative GPI-anchored glucan endo-1,3-β-D-glucosidase); PepJ (AN7962, protease); CatB (AN9339, catalase); and SodA (AN0241, Cu/Zn-SOD). The presence of AbnC, CatB, EglB, PepJ, SodA, and the AN8445 peptidase in the fermentation broth of lactose-containing cultures has already been demonstrated [39]. van Munster et al. [24] found that upregulation of CAZyme genes by carbon stress is accompanied by the secretion of the corresponding proteins in *A. niger* even in the case of carbon starvation. Accordingly, the genes of the proteins detected here were upregulated by the appropriate carbon stress (Table S2) with the following exceptions: We could not detect the upregulation of *eglB* in carbon-starved cultures or the upregulation of *sodA* and AN8445 in lactose-containing cultures (Table S2). However, we could detect the presence of the encoded proteins in the fermentation broth (Table S4, [39]). In the case of SodA and EglC (detected during carbon starvation), as well as of CatB (detected both in carbon-starved cultures and on lactose) (Table S4, [39]), the corresponding genes even showed downregulation by carbon stress (Table S2). These proteins may be accumulated in the cells (in the case of cell-wall-anchored EglC, in the cell wall) on glucose and may be released into the fermentation broth only under stress.

### 3.2. Transcriptome Analyses Revealed Important Differences among the Carbon-Stressed Cultures

Based on principal component analysis, the available carbon source substantially affected the transcriptome of the fungus as expected (Table S5). In the case of 28 genes, transcriptional changes obtained with RNA sequencing were compared with those recorded with RT-qPCR, and a good positive correlation was observed (the Pearson’s correlation coefficient was 0.79) (Table S5).

**Figure 2:**
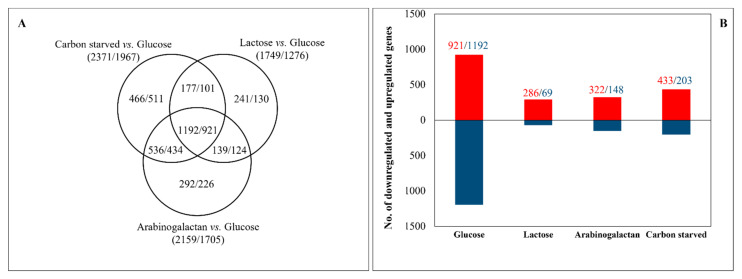
Distribution of carbon-stress-responsive (**A**) and culture-specific (**B**) genes among the cultures. Figures represent the number of upregulated/downregulated genes. Note, the three sets in (**A**) contain carbon-stress-responsive genes, and the intersection of the three sets contains the general stress response genes (using glucose-containing cultures as reference). The upregulated general stress response genes are identical with the downregulated glucose-specific genes and the downregulated general stress response genes are identical with the upregulated glucose-specific genes (**B**).

Carbon-starvation and carbon-limitation stresses changed the expression of many genes (Figure 2A). Earlier studies demonstrated that there is a substantial overlap among the early stress responses of carbon-limited and carbon-starved cultures [24]. This has been explained by the near-starvation of carbon-limited cultures until utilization of the alternative carbon sources is established [24]. Therefore, we took samples at times when utilization of lactose and arabinogalactan had already started according to their growth pattern (Figure 1). Nevertheless, we found substantial overlap among the genome-wide transcriptional changes of the three carbon-stressed cultures: 39% of the carbon-stress-responsive genes showed upregulation in all three carbon-stressed cultures, and 38% showed downregulation (Figure 2A). By definition, these same genes showed culture-specific downregulation and upregulation, respectively, in glucose-rich cultures. They also highly exceeded the number of culture-specific down- and upregulated genes in carbon-stressed cultures (Figure 2B).

Gene set enrichment analyses of carbon-stress-responsive genes suggest that carbon starvation downregulated bulk protein synthesis, several elements of primary metabolism (e.g., glucose utilization, amino acid biosynthesis, steroid synthesis), and the transcription of several stress genes. On the other hand, it upregulated genes involved in cell wall organization, chitin, xylan, and pectin degradation, as well as fatty acid oxidation (Table 2 and Table S6). Replacing glucose with lactose downregulated several elements of primary metabolism (e.g., glucose utilization and steroid synthesis); however, in contrast to carbon-starved cultures, downregulation of amino acid biosynthesis or “ribosome biogenesis” and “translation” was not observed. It upregulated extracellular polysaccharide utilization, including the metabolism of pentoses and hexoses other than glucose (Table 2 and Table S6). Growing on arabinogalactan downregulated glucose utilization, amino acid biosynthesis, and steroid synthesis. As on lactose, downregulation of bulk protein synthesis was not observed. Upregulation of extracellular polysaccharide utilization genes was detected; however, genes involved in pentose or hexose (e.g., galactose, mannose) metabolism were enriched in both the upregulated and downregulated gene sets (Table 2 and Table S6). Gene set enrichment analyses of culture-specific genes suggest that processes related to glucose utilization and growth (e.g., glycolysis, respiration, biosynthesis of steroids, vitamins, cofactors prosthetic groups) were upregulated, while the polysaccharide catabolic process and lipid metabolism (e.g., fatty acid oxidation) were downregulated in glucose-rich cultures relative to all the other cultures (Table S6). In parallel with this, carbon-stressed cultures were characterized by the upregulation of different elements of carbohydrate catabolism and by the downregulation of a few processes mainly related to growth (Table S6).

For further analysis of how carbon-stressed cultures ensure their energy needs, we selected the gene groups presented in Table 3, Table 4 and Table S2:

The *galR* and *galX* genes [44] encoding the transcriptional regulators of lactose utilization genes were upregulated only in lactose-containing cultures (Tables S2 and S5). This was accompanied by the upregulation of the D-galactose oxidoreductive pathway genes [52], and several genes encoding known/putative lactose permeases and β-galactosidases, including the genes of the main β-galactosidase (*lacD*) and lactose permeases (*lacpA*, *lacpB*) (Tables S2 and S5) involved in lactose utilization [45,46] (Table 3, Tables S2 and S5). Upregulation of any Leloir pathway genes was not observed, suggesting that the main route of lactose utilization is the D-galactose oxidoreductive pathway (Table 3 and Table S2).

In arabinogalactan-containing cultures, *araR* [53], but not *galR* or *galX,* was upregulated. Upregulation of the D-galactose oxidoreductive pathway genes, the same as those upregulated on lactose (Table S2), as well as *lacD*, *lacpA*, and *lacpB*, as well as several other known/putative lactose permease and β-galactosidase genes, was also observed. Interestingly, the Leloir pathway genes were even downregulated in the carbon-starved cultures (Table S2), suggesting that this pathway may relate to the synthesis of galactose-containing saccharides rather than galactose degradation. The *lacpB* and *lacD* genes, and some other putative/known β-galactosidase genes, were upregulated even in the carbon-starved cultures (Table 3 and Table S2), which concurs with elevated β-galactosidase activities detected in all carbon-stressed cultures (Table 1).

Glycolysis genes were downregulated by carbon stress (Table 3 and Table S2) but the downregulation of oxidative pentose phosphate shunt and TCA cycle genes was observed only in carbon-starved and arabinogalactan-containing cultures, which showed the smallest growth in our experiments (Figure 1B).

Carbon stress significantly influenced cell wall homeostasis (Table 3 and Table S2). In general, downregulation of synthase, transglycosylase, and regulatory protein genes, as well as upregulation of hydrolase genes, was observed, which concurs with the reduced growth of the cultures (Figure 1B) (Table 3 and Table S2). However, in the case of lactose-containing cultures, which showed the fastest growth out of the three carbon-stressed cultures (Figure 1), the enrichment of the above-mentioned genes in the appropriate gene sets was not significant (Table S2). The cell wall hydrolase genes *engA* (endo-1,3-β-glucanase), *chiB* (endochitinase), and *nagA* (N-acetyl-β-glucosaminidase), which have been demonstrated to be important in the utilization of the cell walls of dead cells (autolytic cell wall degradation [2,54]), were upregulated in carbon-starved and arabinogalactan-containing cultures (Table 3, Tables S2 and S5). These upregulations together with the upregulation of AN1427 (putative N-acetylglucosamine transmembrane transporter gene), AN1428 (putative N-acetylglucosamine-6-phosphate deacetylase gene), and AN1418 (putative glucosamine-6-phosphate deaminase gene), as well as of AN2424 (putative N-acetylhexosaminidase) (Table S2), suggest that autolytic cell wall degradation takes place not only in carbon-starved, but also in slow-growing cultures. Importantly, transcriptional activation of the cell wall integrity pathway was not observed, supporting the view that the cell wall of only dead cells (the so called “empty hyphae”) was degraded and living cells could protect themselves against these hydrolase activities [42]. Previously, we found that melanin production can protect cells against chitinases, including the ChiB chitinase [42]. The *chiB* gene was upregulated not only in carbon-starved and arabinogalactan-containing cultures, but also in cultures growing on lactose (Tables S2 and S5). Not surprisingly, the Ivo cluster, responsible for N-acetyl-6-hydroxytryptophan-type melanin formation, was upregulated (Tables S6 and S3), together with aromatic amino acid metabolism genes (Table 2 and Table S6) in all carbon-stressed cultures. Many cell wall hydrolase genes were downregulated in all three cultures (Table S2). In cultures growing on lactose, cell wall hydrolase genes were even enriched in the downregulated gene set (Table 3 and Table S2). These genes may contribute to the biosynthesis rather than the degradation of the cell wall [49], like the *chiA* gene which was downregulated in all three carbon-stressed cultures (Table S2) and encodes a chitinase involved in cell wall remodeling and maturation during growth [55]. Cell wall hydrolase genes were enriched in the upregulated glucose-specific and carbon-starvation-specific gene sets (Table S2). The two non-overlapping, upregulated gene sets (Table S2) also demonstrate that cells use different sets of hydrolases for cell wall biosynthesis and for cell wall degradation.

Enrichment of upregulated autophagy-related genes was observed only in the carbon-starved cultures (Table S2), which demonstrates that, like autolytic cell wall degradation, autophagy is also an important process that can provide energy sources in the absence of external nutrients.

Carbon stress upregulated extracellular peptidase (protease) genes in all cultures; however, their enrichment in the upregulated gene sets was significant only in carbon-starved and arabinogalactan-utilizing cultures (Table 3 and Table S2). Even in the lactose-containing culture, 10 putative/known extracellular peptidase genes were upregulated, which was accompanied with high protease activities (Table 1). These data demonstrate that—in cultures used for heterologous protein production—any carbon stress can be unfavorable, due to the supposed intensive proteolytic degradation [56].

Upregulated CAZyme genes were significantly enriched in the carbon-stressed cultures (Table 3, Table 4, Figure S1). Some of them are related to the applied carbon source: e.g., enrichment of the upregulated β-galactosidase genes was observed on both lactose and arabinogalactan, while upregulated arabinofuranosidase and endo-arabinosidase, as well as α-galactosidase genes, were enriched in the upregulated stress-responsive genes of arabinogalactan-containing cultures only (Table 3 and Table S2). Upregulation of AN9166 (putative exo-1,6-galactanase [57]) was also observed only on arabinogalactan (Table S2). However, many upregulated CAZyme genes were (putatively) involved in the utilization of carbohydrates that had not been added to the media: Upregulated β-1,4-endoglucanase/cellulase, β-glucosidase, cellobiohydrolase—cellobiosidase genes were enriched on arabinogalactan, while upregulated xylosidase and rhamnogalacturonan utilization genes were enriched in all three carbon-stressed cultures (Table 3, Table 4, and Table S2). The upregulated culture-specific gene sets contained several CAZyme genes in each of the four culture types (Table 4 and Table S2 and Figure S1). The most CAZyme genes (65 genes) were observed in arabinogalactan-containing cultures, surprisingly followed by glucose-rich cultures (29 genes), then by carbon-starved cultures (16 genes), and lastly by lactose-containing cultures (6 genes) (Table 4 and Table S2 and Figure S1). Enrichment of genes belonging to many CAZyme subcategories was observed with arabinogalactan-containing cultures (Table 4 and Table S2 and Figure S1). No enrichment was found with the carbon-starved cultures, suggesting that the CAZyme genes upregulated specifically in these cultures are distributed among several CAZyme subcategories (Table 3 and Table S2). Interestingly, in lactose-containing cultures only α-galactosidase, but not the β-galactosidase genes, showed enrichment. In fact, out of the β-galactosidase genes, only *lacD,* encoding the β-galactosidase, which is essential for lactose utilization, showed significantly higher transcriptional activity on lactose than in all the other cultures (Tables S2 and S5). Glucose-rich cultures were characterized by the enrichment of β-1,4-endoglucanase/cellulase genes (Table 4 and Table S2 and Figure S1).

**Figure 3:**
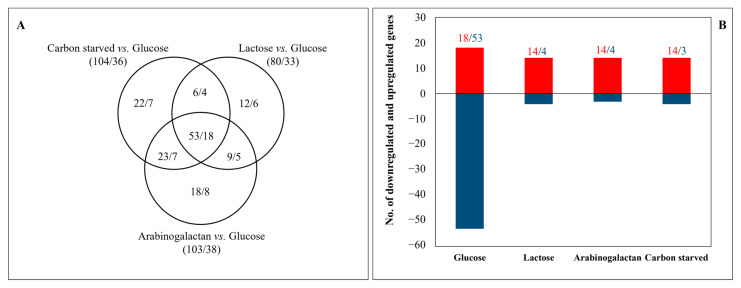
Distribution of carbon-stress-responsive (**A**) and culture-specific (**B**) transcription factor genes among the cultures. Figures represent the number of upregulated/downregulated transcription factor genes.

Transcription factor genes were enriched in the upregulated gene sets of carbon-stressed cultures and in the downregulated glucose-specific gene set (Table S2 and Figure 3). Besides the upregulation of *galR* and *galX* on lactose and *araR* on carbon-starved and arabinogalactan-containing cultures, upregulation of *clrA* responsible for the regulation of cellulase and xylanase production [58], *rhaR* responsible for rhamnose utilization [59], and *brlA* and *xprG* responsible for extracellular peptidase and fungal cell wall hydrolase production [2,54,60] in carbon-stressed cultures are notable (Tables S2 and S5). Many upregulated peptidase, CAZyme, and cell wall genes encode (putative) extracellular enzymes. Accordingly, the gene of HacA [61] transcription factor, responsible for unfolded protein stress response (endoplasmic reticulum stress), was upregulated by carbon stress (Tables S2 and S5).

**Figure 4:**
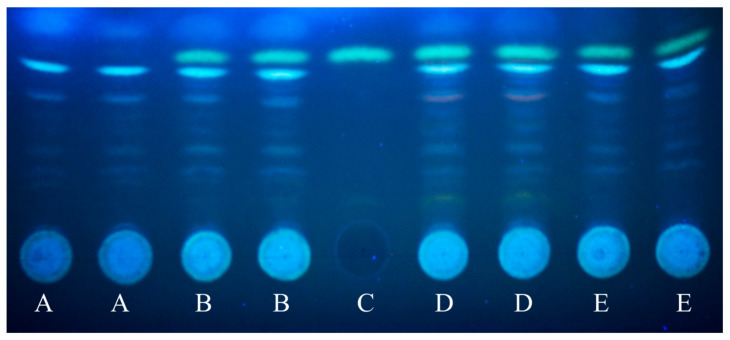
Production of sterigmatocystin by carbon-stressed *A. nidulans* THS30 cultures. A representative photo on the results of a TLC is presented. Mycelial samples were taken 12 h (carbon-stressed cultures) or 4 h (glucose-containing cultures) after the mycelia, grown on glucose, were transferred into fresh media. A—glucose-containing cultures; B—carbon-starved cultures; C—sterigmatocystin standard; D—lactose-containing cultures; E—arabinogalactan-containing cultures.

Since secondary metabolism highly depends on the available carbon source [62], we also evaluated the transcriptional behavior of secondary metabolite cluster genes. These genes were enriched in both the upregulated and the downregulated gene sets of all three carbon-stressed cultures (Table 2 and Table S6). All four cultures had a characteristic set of secondary metabolite cluster genes that showed the highest activity in that culture (Table S6). The upregulated and downregulated clusters showed a similar pattern. The most important clusters upregulated by carbon stress are listed in Table 5. Among them, upregulation of the sterigmatocystin cluster is notable, since all the 26 cluster genes showed upregulation in the three carbon-stressed cultures, and the formation of this mycotoxin was also demonstrated in these cultures with TLC (Figure 4). Clusters characteristically upregulated or downregulated in a culture are summarized in Table S3. It is notable that although the highest number of downregulated clusters was observed on glucose, there were clusters (Microperfuranone cluster, Pkh cluster, and AN3273 cluster) that showed upregulation relative to all the other cultures under this condition (Table 5 and Table S3).

## 4. Discussion

We studied the behavior of four *A. nidulans* cultures differing from one another in the available carbon/energy source. These cultures contained either glucose (as an easily utilizable monosaccharide), or lactose (as a disaccharide, allowing slower growth than glucose does), or arabinogalactan (as a complex polysaccharide providing only slow growth), or did not contain any carbon sources (Figure 1). Since extracellular proteins are accumulated in the fermentation broth, their detectability highly depends on the biomass/culture volume ratio as well as on the duration of accumulation. Moreover, extracellular proteins can persist in the fermentation broth for generations after the transcription of their genes is downregulated, e.g., proteins secreted during the early stress response when both carbon-starved and carbon-limited cultures are starving can remain in the fermentation broth until the late stress response when their production may be downregulated. In order to avoid these limitations, we used a transcriptome-based approach. Nevertheless, we have to keep in mind that changes in the transcription of a gene do not necessarily cause changes in the abundance of the corresponding protein. For example, the aim of a transcriptional upregulation can be to stabilize the protein level, to reduce its fast decrease [63], or simply to prepare for its fast synthesis when (and if) it becomes advantageous.

### 4.1. Carbon-Starved Cultures Produce Many Different “Scouting” Enzymes

Utilization of extracellular polysaccharides is challenging for microbes because they have to identify the polysaccharides present in the environment in order to secrete the appropriate enzyme or enzyme mix for their efficient degradation. This problem is generally solved by secretion of “scouting” enzymes [22,23,24]. These are “ordinary” CAZymes, which do not completely degrade a polymer, but liberate some oligomers/monomers in a cost-efficient manner. These latter compounds (“regulatory molecules”) are recognized by cells, resulting in increased production of all the enzymes needed for the complete and efficient degradation of the polymer [22,23,24].

In our carbon-starved cultures, many CAZyme genes (107 genes) were upregulated relative to glucose-rich cultures. However, only 16 of them were culture specific, i.e., showed higher transcriptional activity in the studied culture than in the other cultures (Table 4 and Table S2 and Figure S1). The CAZyme subgroups studied were not enriched in the upregulated carbon-starvation-specific gene set and most of them were not enriched in the upregulated carbon-starvation-stress gene set, either (Table 3, Table 4 and Table S2). These properties concur well with the “secretion of scouting enzymes” strategy: *A. nidulans*, like *A. niger* [24], secreted several enzymes simultaneously during starvation to search for alternative carbon sources, but did not upregulate complete gene sets needed for the utilization of a polysaccharide.

Interestingly, many of the rhamnoglacturonan degradation genes (12 out of the 16 genes) and almost half of the galacturonan, arabinan, and xylan degradation genes (altogether 25 out of the 54 genes) behaved as scouting enzyme genes (showing upregulation in these cultures), while cellulose degradation genes did not (Table S2, Table 3 and Table 4). Unlike cellulose, the non-cellulosic components of the plant cell wall are quite variable; therefore, their recognition may need several different enzymes. After recognition, however, their efficient degradation may be less problematic than that of cellulose with a partially crystalline structure. This may explain the high ratio of scouting enzyme genes in these groups.

In carbon-starved cultures, utilization of stored compounds (e.g., glycogen or even glutathione [27]), autophagy, and autolytic cell wall degradation (Table 3 and Table S2) [2,42] can provide energy sources. Besides the detection of potential carbon sources, autolytic cell wall degradation also needs intensive production of extracellular enzymes. Not surprisingly, early carbon stress response upregulated a set of ribosome biogenesis genes and ER-specific processes (such as ER to Golgi vesicle transport, protein glycosylation, or ER stress response) [42]. Even in the late stress response studied here, the transcription of *hacA* encoding a transcription factor regulating ER stress response [61] was still upregulated (Tables S2 and S5). Hence, adaptation to carbon stress seems to rely heavily on adapting the behavior of ER. Manipulation of ER activity can lead not only to improved secretion of an enzyme but can also lead to faster adaptation to the applied CAZyme-producing conditions in the fermentation industry. Secreted enzymes and adaptation to carbon stress are both important during fungal infections [8,9,64], which calls attention to antifungal strategies based on disturbing ER activity of the fungus.

### 4.2. Adaptive Prediction Can Be Important in the Regulation of CAZyme Genes on Arabinogalactan

“Adaptive prediction” is a phenomenon commonly used to explain “stress cross protection” in stress biology [25,26]. It means that under stress, cells upregulate stress response elements to cope with the actual stressor and also other elements to prepare for the most likely subsequent stresses. As a consequence, one stressor can increase the tolerance against another stressor. Plant cell wall polysaccharides rarely occur in isolation in the natural habitats of fungi: the presence of one type increases the probability of other types also occurring there. Therefore, it is reasonable to assume that the regulatory molecules formed during the degradation of one type of polysaccharide will upregulate CAZyme genes needed for the recognition and/or the degradation of other possibly co-occurring polymers as well (“cross-upregulation”).

The arabinogalactan used in our study consists of a β-1,3-D-galactopyranosyl polymer as a backbone with side-chains including α-L-arabinofuranosyl (at C6), β-1,6-L-galactobiosyl (at C4 or C6), and 4-O-(α-L-arabinofuranosyl)-β-D-galactopyranosyl (at C6) units (https://www.megazyme.com/arabinogalactan-larch-wood; 10 December 2021). Not surprisingly, several genes encoding enzymes potentially involved in the degradation of this polymer were upregulated, including galactosidase and arabinofuranosidase genes (Table 3, Table 4 and Table S2). Interestingly, the genome of *A. nidulans* does not encode any orthologue of *A. flavus* Af3G β-1,3-endogalactanase [65]. We only observed upregulation of AN9166 (a putative exo-1,6-galactanase gene), but not of *galA* (β-1,4-endogalactanase) (Table S2). Galactose and arabinose were most likely utilized via the D-galactose oxidoreductive pathway and the overlapping pentose catabolism pathway [53], respectively (Table S2). Growing on arabinogalactan did not upregulate autophagy genes, suggesting that energy production shifted towards the utilization of arabinogalactan components; however, the transcription of genes involved in autolytic cell wall degradation (e.g., *chiB*, *engA*, *nagA*) was still high (Table S2). Interestingly, arabinogalactan-containing cultures showed bulk upregulation of genes involved in, or putatively involved in, xylan, galacturonan, rhamnogalacturonan, or cellulose utilization as well (Table 3, Table 4 and Table S2). This upregulation cannot be explained by the “secretion of scouting enzymes” strategy: Many of the upregulated genes showed significantly higher transcriptional activity on arabinogalactan than in all the other cultures, and in many subgroups (α-glucosidases, β-glucosidases, β-1,4-endoglucanases, cellobiohydrolase—cellobiose dehydrogenases) more genes showed upregulation on arabinogalactan than in carbon-starved cultures (Table 4 and Table S2 and Figure S1). This property of arabinogalactan-containing cultures could be explained by postulating a cross-upregulation effect of some of the regulatory molecules liberated from arabinogalactan. Similar cross-upregulation between some CAZyme groups has already been documented: Production of certain cellulolytic enzymes is induced in the presence of xylose in *A. niger* [66], or on arabitol and xylans in *Trichoderma reesei* [67]. Moreover, since lactose as a β-galactoside disaccharide is structurally more similar to the oligomers formed during the degradation of galactose-containing polysaccharides than to those released during cellulose degradation, the induction of cellulases by lactose in *T. reesei* [68] and in *Acremonium cellulolyticus* [69] can also be the consequence of cross-upregulation.

### 4.3. High Lactose Concentration May Activate Strategies to Control the Cheaters

Extracellular degradation of polysaccharides leads to formation of public goods, i.e., extracellular mono- and oligosaccharides freely available for any cells in the vicinity. Since cheaters use public goods but—by definition—do not invest in enzyme secretion, they can easily increase in population [14]. The successful management of this problem is complex. Different mechanisms, including the use of cell-surface-attached enzymes to limit the diffusion of the liberated compounds away, improved uptake of the liberated nutrients, or development of spatial structures to separate enzyme producers from cheaters can be used [14]. Extracellular degradation of biopolymers is commonly controlled by feedback inhibition and repression [4,70]. These negative feedback regulatory mechanisms, together with the quick utilization of the liberated molecules, control the size of public goods and limit the amount of liberated compounds diffused away from enzyme producers. Hence, negative feedback regulation can play an important role in the restriction of cheaters.

Free lactose is rare in nature (commonly occurring in the milk of mammals); however, both the β-galactoside linkage (e.g., in xyloglucans, rhamnogalacturonans, and arabinogalactan-proteins) and α-galactoside linkage (e.g., in galactomannans, galactoglucomannans, and extensins) are common in plant cell wall saccharides [11]. Galactose is also part of the galactomannan (as galactofuran side-chains) and galactosaminogalactan (as α-1-4-linked galactose and N-acetylgalactosamine residues) components of the fungal cell wall [71]. Not surprisingly, the genome of *A. nidulans*, similarly to those of many other fungi, contains several α- and β-galactosidase as well as a few galactanase genes (AspGD; Table S2) to hydrolyze these polysaccharides and/or the oligo- and disaccharides liberated during their degradation.

Lactose-containing cultures were characterized by the upregulation of genes directly involved in lactose utilization, such as *lacD* β-galactosidase, *lacpA,* and *lacpB* lactose permeases [45,46], as well as the D-galactose oxidoreductive pathway [53] (Table S2). Only 81 CAZyme genes were upregulated on lactose, and all but one of them were also upregulated either on arabinogalactan and/or during carbon starvation (Table 4 and Table S2 and Figure S1). Cells growing on lactose upregulated fewer extracellular peptidase and (fungal) cell wall hydrolase genes than in the two other carbon-stressed cultures (Table S2). Interestingly β-galactosidase genes other than *lacD* were also upregulated on lactose; moreover, several α-galactosidase genes were also upregulated (Table 4 and Table S2 and Figure S1). Among the encoded enzymes, LacA is known to hydrolyze lactose but also hydrolyze the terminal β-1,3 and β-1,4 galactofuranosyl residue from oligosaccharides [72]. The *lacA* gene, unlike *lacD*, encodes an N-terminal signal peptide sequence suggesting extracellular use of the enzyme (AspGD). Most likely, the majority of the upregulated galactosidase genes are involved in the utilization of galactose-containing compounds other than lactose. Among the genes playing a role in the degradation of galactose-containing polymers (xyloglucan and rhamnogalacturonan degradation genes as well as galactanase, arabinofuranosidase, and endo-arabinosidase genes), fewer were upregulated on lactose than in the two other carbon-stressed cultures (Table 4 and Table S2 and Figure S11). The number of upregulated genes related to the degradation of compounds not containing galactose (β-1,4-endoglucanase, β-glucosidase, cellobiosidase, and cellobiose dehydrogenase genes) was 11 on lactose, i.e., more than those recorded in carbon-starved (8 genes) and less than in arabinogalactan-containing cultures (19 genes) (Table 4 and Table S2 and Figure S1). These changes together suggest that a high lactose concentration may have imitated a situation where galactose-containing polysaccharides were present in the environment and their degradation was so efficient that the galactose-containing oligomers started to accumulate extracellularly. In this situation, the importance of searching for alternative nutrients, upregulating genes involved in the degradation of possibly co-occurring polysaccharides, activating autophagy, or maintaining intensive autolytic cell wall degradation is reduced. However, cells need to keep the amount of public goods at an appropriate (not too high) level to prevent the rise of cheaters. Therefore, the rate of utilization and the rate of liberation of any potential public goods should be balanced. This leads to a CAZyme profile on lactose where more CAZyme genes involved in the degradation of identified/predicted polysaccharides are upregulated than in carbon-starved cultures, but less than on arabinogalactan (Table 4 and Table S2 and Figure S1).

It is very likely that the effect of lactose highly depends on its concentration and that it acts as a dual regulatory molecule. At low levels, it can upregulate genes involved in (galactose-containing and co-occurring) polysaccharide degradation to enhance their utilization as found in other species [68,69], while at high concentrations it may downregulate them to control oligomer concentration in the environment. Similar behavior was found with xylose which can either upregulate (at low concentration) or downregulate (at high concentration) several xylanolytic and cellulolytic genes in *A. niger* [73]. Moreover, this dual regulation of cellulolytic enzyme production was observed in *A. terreus* with xylose, cellobiose, and even with glucose [74]. In the case of *Saccharomyces cerevisiae* invertase, upregulation of the corresponding *suc2* gene by low concentration of glucose was also observed alongside glucose repression at high glucose concentrations [75].

### 4.4. High Glucose Concentration Can Lead to the “Tragedy of the Commons” Scenario

Garrett Hardin’s “tragedy of the commons” scenario occurs when the strategy of public goods utilization is beneficial for individuals but not for the community, since it leads to total depletion of public goods. It is commonly explained by the example of a public pasture used by various herdsmen. The best (self-)interest of each herdsman is in adding new animals to their own herd, since the negative consequences of overgrazing are shared evenly, but the benefits of extra animals will accrue to their owners individually. However, the addition of new animals to the different herds, continuing according to this principle of self-interest, will ultimately result in the complete destruction of the pasture by overgrazing [14]. In the case of extracellular polysaccharide utilization, the “tragedy of the commons” scenario can be prevented if microbes balance public goods formation and utilization. Since the best interest of each cell is using as much public goods as possible and not investing in further enzyme secretion to keep the balance (facultative cheating [76]), the fast depletion of public goods is predicted, unless cheaters are controlled.

Although free glucose is not as rare as lactose in the environment, it is not an abundant molecule in the soil or many other habitats of *Aspergillus* species. The majority of glucose occurs as monomers of different α- and β-glucans. Regarding the plant cell wall, cellulose, mixed-linkage glucan, xyloglucan, and glucomannan are the most common glucose-containing compounds [11], while in the case of the fungal cell wall, β-1,3- and α-1,3-glucans are notable [49].

In cultures growing on glucose, glycolysis genes were upregulated, while autophagy and autolytic cell wall degradation genes were downregulated relative to the carbon-starved cultures (Table S2). Extracellular peptidase genes as well as CAZyme genes also showed low transcriptional activity (Table S2). Interestingly, some CAZyme genes reached the highest transcriptional activity on glucose: Altogether, 29 genes had significantly higher activity in these cultures compared to all three carbon-stressed cultures (Table 4 and Table S2 and Figure S1). Among them, *agdB* (α-glucosidase) [77], *bglJ* (β-glucosidase) [57], *xgcA* (xyloglucanobiohydrolase) [78], as well as four putative β-1,4-endoglucanase genes (AN1041, AN6786, AN7891, and AN8068), are notable. These genes encode enzymes that are involved, or putatively involved, in the degradation of glucose polymers. Importantly, we could detect low cellulase activity (but not β-glucosidase activity) in the fermentation broth of glucose-containing cultures (Table 1). Induction of both endoglucanase and β-glucosidase activities was recorded on (low concentration of) glucose in *A. terreus* [74], and β-glucosidase secretion on glucose was also detected in different *Aspergillus* species [42,79]. The above-mentioned characteristics of cultures growing on glucose (e.g., the numerous upregulated culture-specific genes involved in β-glucan degradation) resemble those observed with lactose. We can assume that a high glucose concentration also imitates a situation for *A. nidulans* when it grows on a polysaccharide and its degradation is so efficient that monomers have accumulated in the environment. Therefore, cultures enhanced their glucose utilization, controlled glucose liberation, and downregulated processes aimed at searching for alternative nutrients or to degrade predicted polysaccharides other than glucans; these cultures also downregulated autophagy or autolytic cell wall degradation. Downregulation (low transcriptional activity) of genes encoding extracellular enzymes (e.g., peptidases or plant and fungal cell wall hydrolases) on glucose was more obvious than on lactose, suggesting that cheating was a more beneficial strategy on glucose than on lactose. This may be explained by noting that cells can utilize glucose much faster than lactose. Preventing the depletion of glucose as public goods would thus need very intensive glucan degradation, and the high enzyme activity needed for it represents a high cost that is not favorable for cooperation. This leads easily to the “tragedy of the commons” scenario and the rise of facultative cheating.

A good strategy for (facultative) cheaters is investing in fast vegetative growth. Growing, as an autocatalytic process, needs high levels of glucose as energy and a carbon source itself; in addition, the newly formed cells will also utilize glucose for their further growth. As a consequence, fast growing allows cells and their progeny to utilize more from public goods than other, slower-growing cells. Not surprisingly, genes related to the utilization of glucose and vegetative growth, including glycolysis genes, aerobe respiration genes, 2-oxocarboxylic acid metabolism, and ergosterol biosynthesis genes, as well as genes involved in the biosynthesis of vitamins, cofactors, and prosthetic groups, and additionally those involved in nitrogen, sulfur, and selenium metabolism or cell wall homeostasis were enriched in the upregulated glucose-specific gene set (Tables S2 and S6). Antioxidant enzyme genes were also enriched in the upregulated glucose-specific gene set (Table S2). This concurs well with aerobic utilization of glucose leading to the formation of reactive oxygen species, and supports the idea that fast growth based on an aerobic metabolism can be dangerous for microbes [80]. The downregulated glucose-specific gene set was even bigger than the upregulated one (Figure 2B). It was enriched with extracellular peptidase and CAZyme genes (other than β-1,4-endoglucanase genes) (Table S2). Moreover, the highest number of downregulated secondary metabolism clusters was also observed on glucose (Table S3). The enrichment of transcription factor genes in this set (Figure 3B and Table S2) (even though some of them may encode a negatively acting regulator) suggests that many processes active in carbon-stressed cultures were downregulated on glucose. In summary, cells did not only upregulate many genes on glucose to enhance glucose utilization but also downregulated numerous unnecessary genes/processes (including many elements of secondary metabolism) which could otherwise reduce their growth rate and hence the quick utilization of public goods.

Out of the four cultures we studied, the glucose-rich cultures contained the most genes that reached their highest or lowest transcriptional activity under the studied conditions (Figure 2B). This is important because when we compare two transcriptomes, the obtained difference necessarily depends on both of them. Previously, we suggested that the recorded (stress) response equally characterizes how the cells have adapted to the conditions before the (stress) treatment and how they are trying to adapt to the new conditions [81]. It is also true when the consequences of more than one treatment are compared. The size and composition of gene sets upregulated or downregulated in all the treatments (core stress response genes) are highly dependent on the chosen reference cultures. In our study, if we choose the glucose-containing cultures as “stress-free” reference cultures, we can obtain a large core stress response gene set (Figure 2). The huge number of co-regulated genes suggests that carbon starvation and limitation stress responses are similar. However, it also means that the cultures growing on glucose are quite different from the other cultures studied. On the other hand, if we select, for instance, carbon-starved cultures as the reference, the difference among the “carbon-source-induced stress” responses would be much smaller, and the nature of the core stress response genes would also be very different. Finding a good reference condition has primary importance in transcriptome-based studies and our selection should depend on the question(s) we want to answer. In the case of the *Aspergillus* species, choosing glucose-rich cultures as a reference can be practical in general, but is usually not a good choice if we want to understand how they respond to different treatments in their natural habitats or in the human body [26,63]. In contrast, in the case of the *Saccharomyces* species, where glucose feast is common in their sucrose-rich habitats due to their high periplasmic invertase activity [75], studying stress responses on glucose seems to be not only convenient due to the intensive growth, but reasonable as well.

### 4.5. Concluding Remarks

*A. nidulans,* as a typical soil-borne fungus, usually grows on decaying plant materials. Regulation of the several hundred CAZyme and other genes needed for the efficient degradation of plant biopolymers is a complex process. “Regulatory molecules” formed during the degradation of these polymers play a central role [4,12,13,70]. They are important in the recognition of the compounds present (see “secretion of scouting enzymes” by carbon-starved cultures), they can have cross-upregulatory activity (see “adaptive prediction of the available biopolymers” on arabinogalactan), and at high concentrations, they can downregulate genes which can be important in the control of cheaters (see lactose-containing cultures), or can even lead to the rise of facultative cheaters (see glucose-rich cultures). Detailed investigation of both the molecular and behavioral ecological aspects of this complex regulatory network in the future can help us to understand CAZyme secretion of fungi better, which may enhance their industrial application. For example, systematic recording of CAZyme co-regulations can draw our attention to less-studied hydrolases which can enhance the degradation of plant cell wall polysaccharides in the industry as auxiliary enzymes. Studying fungal strategies based on adaptive prediction for polysaccharide degradation can lead to the discovery of new regulatory molecules. Knowledge of these molecules or understanding their dual (up- and downregulatory) nature can promote the development of new, cost-efficient fermentation processes for CAZyme production.

Regulation of CAZyme gene expression is a good example of cells initially recognizing a stress (change), but not yet “knowing” how to adapt to it. Hence, as a first step they give a “scouting response” and use the collected information to drive further steps in the right direction. It is reasonable to assume that stress responses other than the carbon-limitation stress response may also involve different kinds of “scouting elements” to direct the response in the appropriate direction. This would explain why fungi can commonly adapt even with artificially modified genetic backgrounds, and even to very different artificial environments.

## Figures and Tables

**Table 1:** Enzyme activities of the studied *A. nidulans* THS30 cultures.

	Glucose	Lactose	Arabinogalactan	Carbon-Starved
Intracellular enzyme activities				
SOD (U mg^−^^1^ protein)	81 ± 10 ^a,s^	97 ± 15 ^a,s^	156 ± 21 ^g,l,s^	210 ± 30 ^g,l,a^
Catalase (kat kg^−^^1^ protein)	2.2 ± 0.3	1.6 ± 0.3	1.7 ± 0.2	1.7 ± 0.2
Nitrate reductase (mkat kg^−^^1^ protein)	1.40 ± 0.09 ^l,a,s^	1.09 ± 0.05 ^g,a,s^	0.62 ± 0.12 ^g,l^	0.56 ± 0.15 ^g,l^
Glutathione reductase (mkat kg^−^^1^ protein)	9.3 ± 0.3 ^l,a,s^	6.4 ± 0.8 ^g,a^	4.0 ± 0.5 ^g,l,s^	5.3 ± 0.6 ^g,a^
β-Galactosidase (µkat kg^−^^1^ protein)	<0.1 ^l,a,s^	10.2 ± 1.1 ^g,a,s^	3.7 ± 0.3 ^g,l,s^	0.6 ± 0.02 ^g,l,a^
Extracellular enzyme activities				
SOD (U mL^−^^1^)	4.3 ± 1.1 ^l,a,s^	9.7 ± 1.5 ^g^	8.1 ± 1.0 ^g^	9.3 ± 0.9 ^g^
Catalase (µkat mL^−^^1^)	<8 ^l,a,s^	11 ± 1 ^g,a,s^	16 ± 1 ^g,l,s^	52 ± 10 ^g,l,a^
β-Galactosidase (nkat mL^−^^1^)	<0.006 ^l,a^	0.012 ± 0.001 ^g,a,s^	0.018 ± 0.002 ^g,l,s^	<0.006 ^l,a^
β-Glucosidase (nkat mL^−^^1^)	<0.006 ^l,a,s^	0.09 ± 0.02 ^g,a^	0.18 ± 0.01 ^g,l,s^	0.11 ± 0.02 ^g,a^
Cellulase (nkat mL^−^^1^)	0.7 ± 0.1 ^l,a,s^	2.5 ± 0.3 ^g,a,s^	1.2 ± 0.3 ^g,l,s^	<0.3 ^g,l,a^
Chitinase (U mL^−^^1^)	<0.1 ^l,a,s^	0.6 ± 0.2 ^g,a,s^	1.1 ± 0.2 ^g,l^	1.5 ± 0.3 ^g,l^
Proteinase (U mL^−^^1^)	<0.1 ^l,a,s^	3.1 ± 0.3 ^g,a^	0.8 ± 0.3 ^g,l,s^	2.7 ± 0.3 ^g,a^
γGT (nkat mL^−^^1^)	<0.01 ^l,a,s^	0.12 ± 0.02 ^g,a,s^	0.84 ± 0.05 ^g,l,s^	0.35 ± 0.05 ^g,l,a^

Table contains mean ± SD vales calculated from three biological replicates. ^g, l, a, s^—Significant difference (Student’s *t*-test, *p* < 0.05) from the data of cultures containing glucose, lactose, arabinogalactan, or no carbon source, respectively.

**Table 2:** Selected significantly enriched FunCat, GO, and KEGG pathway terms of the carbon-stressed *A. nidulans* cultures.

Comparison ^a^	Significantly Enriched Terms	
	In Upregulated Gene Set	In Downregulated Gene Set
Lactose vs. Glucose (1749/1276)	glycerolipid metabolism, fatty acid metabolism, polysaccharide catabolic process, mannan catabolic process, xylan catabolic process, cellulose catabolic process, arabinose metabolic process, D-xylose metabolic process, xylulose metabolic process, galactose metabolic process, fructose and mannose metabolism; alpha-galactosidase activity, secondary metabolism, sterigmatocystin biosynthetic process, metabolism of melanins, phenylalanine metabolism, tryptophan metabolism, tyrosine metabolism,	glycolytic process, gluconeogenesis, respiration, mitochondrion, steroid biosynthesis, biosynthesis of vitamins, cofactors, and prosthetic groups, biosynthesis of secondary metabolites
Arabinogalactan vs. Glucose (2159/1705)	glycerolipid metabolism, oxidation of fatty acids, cell wall organization, extracellular polysaccharide degradation, pectin catabolic process, xylan catabolic process, glucan catabolic process, cellulose catabolic process, mannan catabolic process, pentose-phosphate pathway, arabinose metabolic process, D-xylose metabolic process, galactose metabolism, beta-glucosidase activity, secondary metabolism, sterigmatocystin biosynthetic process, phenylalanine metabolism, tryptophan metabolism, tyrosine metabolism	glycolysis and gluconeogenesis, respiration, citrate cycle (TCA cycle), mitochondrion, biosynthesis of amino acids, biosynthesis of vitamins, cofactors, and prosthetic groups, steroid biosynthesis, pentose phosphate pathway, fructose and mannose metabolism, galactose metabolism, biosynthesis of secondary metabolites, response to stress, cellular response to osmotic stress
Carbon-starved vs. Glucose (2371/1967)	glycerolipid metabolism, oxidation of fatty acids, peroxisome, cell wall organization, extracellular polysaccharide degradation, chitin catabolism, xylan catabolic process, pectin catabolic process, secondary metabolism, sterigmatocystin biosynthetic process, metabolism of melanins, phenylalanine metabolism, tryptophan metabolism, tyrosine metabolism	translation, ribosome biogenesis, nucleotide-sugar metabolism, glycolysis and gluconeogenesis, respiration, Fe/S binding, mitochondrion, cellular amino acid biosynthetic process, cysteine biosynthetic process, sulfate assimilation, steroid biosynthesis, biosynthesis of vitamins, cofactors, and prosthetic groups, heavy metal ion transport (Cu^2+^, Fe^3+^, etc.), biosynthesis of secondary metabolites, oxidative stress response, heat shock response, cellular response to osmotic stress

^a^—Numbers indicated in parentheses represent the number of upregulated and downregulated genes, respectively in the given comparison. The full list is available in Table S6.

**Table 3:** Summary of the regulation of the selected gene groups in carbon-stressed cultures.

	Behavior of the Genes ^a^		
	Lactose vs. Glucose	Arabinogalactan vs. Glucose	Carbon-Starved vs. Glucose
Lactose utilization genes	up	up	-
β-galactosidases—lactose permeases	up	up	up
Leloir pathway	-	-	down
D-galactose oxidoreductive pathway	up	-	-
Glycolysis genes	down	down	down
Oxidative pentose-phosphate shunt genes	-	down	down
TCA cycle genes	-	down	down
Autophagy genes	-	-	up
Cell wall genes	down	down	both
Synthases, transglycosylases, and regulatory proteins	-	down	down
Hydrolases	down	up	up
Extracellular peptidase genes	-	up	up
CAZyme genes	both	up	up
α-Glucosidase	-	-	-
β-Glucosidase	-	up	-
β-1,4-Endoglucanase	-	up	-
Cellobiohydrolase—cellobiose dehydrogenase	-	up	-
α-Galactosidase	up	up	-
β-Galactosidase	up	up	-
Arabinofuranosidase and endo-arabinosidase	-	up	-
Xylanase	-	-	-
Xylosidase	up	up	up
Mannan degradation	-	up	-
Galacturonan degradation	-	up	up
Rhamnogalacturonan degradation	up	up	up
Antioxidant enzyme genes	-	-	down
Squalene—ergosterol pathway genes	down	down	down
Ribosome biogenesis genes	-	-	down
Mitotic cell cycle genes	-	-	-
Transcription factor genes	up	up	up

^a^—“up”, “down”, and “both” stand for significant enrichment in the upregulated, in the downregulated, and in both the upregulated and downregulated gene sets. When no significant enrichment was observed, the “-” symbol was used. Further details on the behavior of the gene groups are available in Table S2.

**Table 4:** Transcriptional behavior of CAZyme genes in the four types of *A. nidulans* cultures.

Group ^a^	Glucose ^b^	Lactose ^b^	Arabinogalactan ^b^	Carbon-Starved ^b^
CAZyme genes (317)	77 (29)	81 (6)	138 (62)	107 (16)
α-Glucosidases (9)	2 (1)	0 (0)	4 (2)	1 (1)
Cellulolytic enzymes (32)	10 (5)	11 (0)	19 (14)	8 (1)
β-Glucosidases (13)	3 (1)	4 (0)	8 (5)	5 (0)
β-1,4-Endoglucanases (14)	5 (4)	5 (0)	7 (5)	2 (1)
Cellobiohydrolases—cellobiose dehydrogenases (5)	2 (0)	2 (0)	4 (4)	1 (0)
Galactoside-degrading enzymes (16)	1 (0)	10 (4)	11 (4)	5 (0)
α-Galactosidases (7)	1 (0)	5 (3)	5 (1)	2 (0)
β-Galactosidases (7)	0 (0)	5 (1)	6 (2)	3 (0)
Galactanases (2)	0 (0)	0 (0)	0 (1)	0 (0)
Arabinofuranosidases and endo-arabinosidases (14)	4 (1)	5 (0)	10 (7)	6 (0)
Xylanolytic enzymes (24)	2 (0)	17 (1)	19 (13)	14 (2)
Xylanases (5)	1 (0)	2 (0)	3 (3)	2 (1)
Xylosidases (12)	1 (0)	10 (1)	11 (8)	7 (0)
Further xylan-degrading enzymes (7)	0 (0)	5 (0)	5 (2)	5 (1)
Mannan degradation enzymes (19)	4 (1)	5 (0)	9 (4)	6 (0)
Xyloglucan degradation enzymes (3)	1 (1)	1 (0)	2 (1)	2 (0)
Pectinolytic enzymes (40)	4 (1)	12 (1)	24 (7)	23 (3)
Galacturonan degradation enzymes (24)	4 (1)	4 (0)	12 (4)	11 (2)
Rhamnogalacturonan degradation enzymes (16)	0 (0)	8 (1)	12 (3)	12 (1)

^a^—Figures presented in parentheses after the name of the gene group indicate the number of the related genes. ^b^—Table contains the number of upregulated genes defined as follows: In the case of carbon-stressed cultures, upregulation was studied relative to glucose-containing cultures. In the case of the glucose-containing culture, upregulation was studied relative to any carbon-stressed cultures (i.e., genes downregulated by any carbon stress). Figures presented in parentheses indicate the number of culture-specific genes. Further data on the transcriptional behavior of CAZyme genes are available in Table S2. See also Figure S1.

**Table 5:** List of selected secondary metabolism cluster genes upregulated by carbon stress in *A. nidulans*.

Cluster ^a^	Lactose vs. Glucose	Arabinogalactan vs. Glucose	Carbon-Starved vs. Glucose
	Number of Upregulated/Downregulated Cluster Genes		
Sterigmatocystin cluster (26)	26/0	26/0	26/0
Monodictyphenone cluster (12)	12/0	12/0	11/0
AN8105 cluster (10)	8/1	9/1	8/1
Pkb cluster (9)	6/0	7/0	8/0
Pkg cluster (6)	6/0	3/0	6/0
Emericellamide cluster (5)	0/0	5/0	1/1
Terriquinone cluster (5)	5/0	5/0	5/0
AN1680 cluster (4)	1/0	4/0	4/0
Penicillin cluster (3)	0/1	3/0	3/0
Ivo cluster (2)	2/0	2/0	2/0
AN9129 cluster (2)	2/0	2/0	2/0
AN9314 cluster (2)	2/0	2/0	2/0

^a^—Only upregulated clusters where all (in the case of clusters with ≤8 genes) or all but 1 gene (in the case of clusters with >8 genes) showed simultaneous upregulation in at least one comparison are presented. The number of genes involved in the cluster is indicated in parentheses. Further data on the transcriptional behavior of the secondary metabolite cluster genes are available in Table S3.

## Data Availability

Transcriptome data sets are available in the Gene Expression Omnibus database (GEO; http://www.ncbi.nlm.nih.gov/geo/; 10 December 2021) with the following accession number: GSE189016.

## Supplementary Materials

The following are available online at https://www.mdpi.com/article/10.3390/jof8010079/s1, Figure S1: Effects of carbon sources on the upregulation of selected CAZyme gene groups. Table S1: RT-qPCR primer pairs used in the study. Table S2: RNAseq data of selected gene groups. Table S3: RNAseq data of secondary metabolite cluster genes. Table S4: Secretome data of selected proteins. Table S5: RT-qPCR data of selected genes and principal component analysis of the RNAseq data. Table S6: Results of the gene set enrichment analyses.
