# Supplementary material for: Strategies Shaping the Transcription of Carbohydrate-Active Enzyme Genes in Aspergillus nidulans
Source: J Fungi (Basel). 2022 Jan 14;8(1):79. doi: 10.3390/jof8010079 (PMC8780418; doi:10.3390/jof8010079)
Supplement: Supplementary file 1 [file jof-08-00079-s001.zip › jof-1527581-supplementary/Figure S1.pdf]

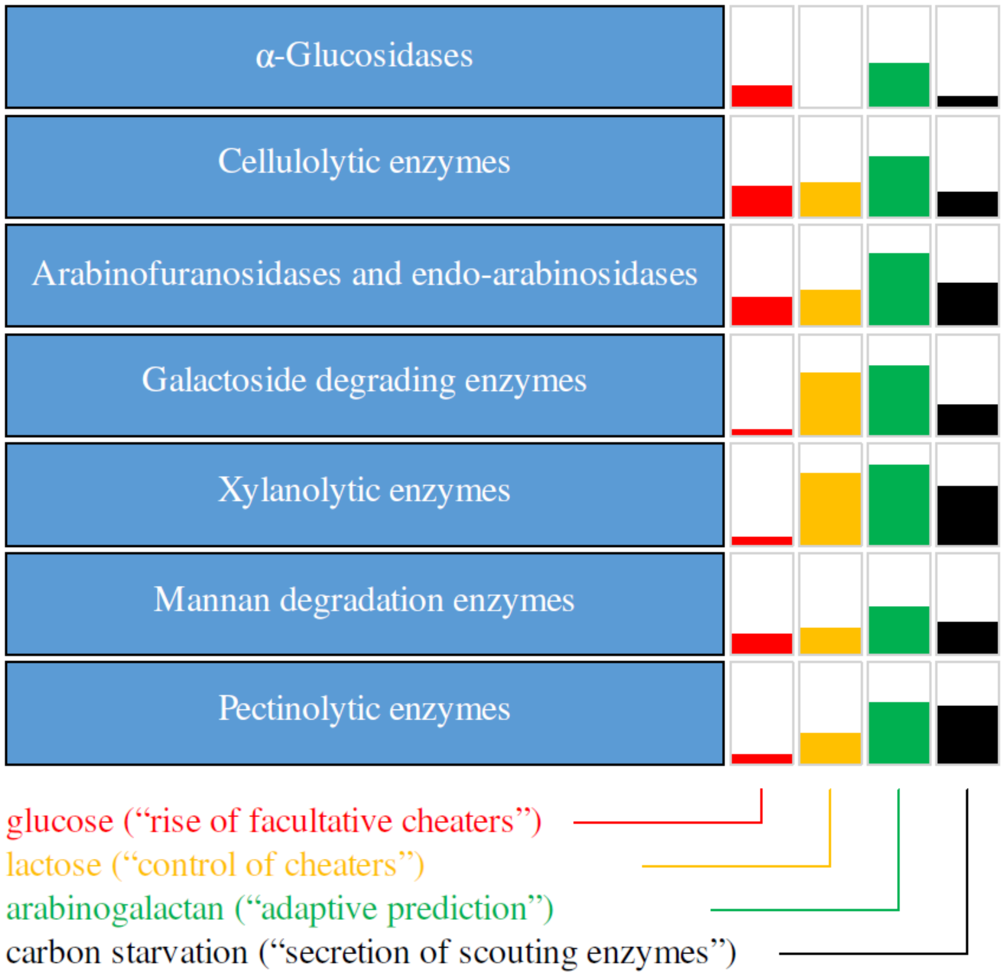

Figure S1 Effects of carbon sources on the upregulation of selected CAZyme gene groups Partially filled boxes on the right show the ratio of upregulated genes in the enzyme group under the color-coded culture types. See also Table 4.
