# Supplementary material for: Strategies Shaping the Transcription of Carbohydrate-Active Enzyme Genes in Aspergillus nidulans
Source: J Fungi (Basel). 2022 Jan 14;8(1):79. doi: 10.3390/jof8010079 (PMC8780418; doi:10.3390/jof8010079)
Supplement: Supplementary file 1 [file jof-08-00079-s001.zip › jof-1527581-supplementary/Table S1.pdf]

Table S1 RT-qPCR primer pairs used in the study.

Gene	Foward primer	Reverse primer
AN0241 (*sodA*)	5’-CTTCCACATCCACCAGTTC-3’	5’-CAGCGTTACCAGTCTTCTTG-3’
AN0399 (*nrtB*)	5’-GCGGGACAGATTGAGAGTTC-3’	5’-AGAGGGAAGTTGAGGAGATAG-3’
AN0423 (*xyrA*)	5’-ATTGGGTAAGGCGACTATCC-3’	5’-AAGGTATGGGTGGTGCTC-3’
AN0756 (*lacA*)	5’-GCTGCTGGTGTTGCTCTTAC-3’	5’-ACTCTCCTGGCTTTCCTTCG-3’
AN0942 (*ladA*)	5’-CCTTTACTCTCTTCACCTCTCC-3’	5’-TCACCACTCTTCACCTCTTCG-3’
AN0973 (*brlA*)	5’-CGACTTTCTCTCTGGATACGATG-3’	5’-CTGGTGACGGTAGTTGTTGTTG-3’
AN1006 (*niaD*)	5’-TATGTCGTCCCAAAACCCG-3’	5’-TTATTCTTCGTCCGCCTCC-3’
AN1414 (*xprG*)	5’-CGATGCCAGTATATCCGTG-3’	5’-GTCAGTTCTGCTCCTTGC-3’
AN1812 (*jlbA*)	5’-ATCTCTCCCCATTCCTTCAGC-3’	5’-GTCGTCCGCCGTAGTTGTG-3’
AN2463 (*lacF*)	5’-CGGGCACACTTCTACTCATAC-3’	5’-TACGACACATTTGGCGGCG-3’
AN2814 (*lacpB*)	5’-CGTCTCCTTCTGCTGTGC-3’	5’-GTGCTTGCCGTTGCGATG-3’
AN3199 (*lacpA*)	5’-CGATTCCAGCCGTCTTCTTC-3’	5’-AGCCGTAAACAACCCAGC-3’
AN3201 (*lacD*)	5’-GCTGCTGAACGGGGATTG-3’	5’-GGGGAACGGGAAGATGAC-3’
AN3581 (*trxR*)	5’-TGGCAGAACGGTATCAGCG-3’	5’-GCGGACAAGCACGGTAACG-3’
AN4871 (*chiB*)	5’-TGGTCACCAGGCGAATCTC-3’	5’-CGGGACGAAGGATCATACG-3’
AN4957 (*galE*)	5’-CGCTGAGGTTTATGCCACTG-3’	5’-CACTGCTGTAGGGAGGAC-3’
AN6388 (*lacE*)	5’-ATGTCGTGCGTCTGCCTC-3’	5’-GTTCTCTTCCCCTTGCTGC-3’
AN6542 (*actA*)	5’-GAAGTCCTACGAACTGCCTGATG-3’	5’-AAGAACGCTGGGCTGGAA-3’
AN6700	5’-CTATTCCCGAGCAAGTTC-3’	5’-TGATGTTCCTGACGATGGC-3’
AN6838 (*tubC*)	5’-CGGAAACTCGCCGTCAATAT-3’	5’-GGGCAAACCCGACAATAA-3’
AN8218 (*trxB*)	5’-TCACCTCAATCGTCCCTG-3’	5’-TGCTCGTATCCGTCACAC-3’
AN8445	5’-TTGAAGCCACGACAATGAC-3’	5’-AGATGCCTACGATACCAG-3’
AN8692 (*prxA*)	5’-CTGGACTGAGGAGAAGGG-3’	5’-CAAGGACGGCAACAACATCG-3’
AN9064 (*xdhA*)	5’-TCTATTGTGCTGTGCGGTAG-3’	5’-TCCCAAGGTCATTCTCTGC-3’
AN9339 (*catB*)	5’-CCGAGCCCGACAACACTTAC-3’	5’-GTTCAGCGACGACAATGACG-3’
AN9397 (*hacA*)	5’-AGACGAAGAATGTGGTGGC-3’	5’-ACGCTGAAGAAGGAACTGG-3’
AN10220 (*ccp1*)	5’-GCGACCAAGAACCAAGACC-3’	5’-AACCAACAGGCGGAAAAACTC-3’
AN10543 (*galX*)	5’-TGACGGGATGGGATTCTTGAC-3’	5’-GGGATACGCAGGGTGATAG-3’
AN10550 (*galR*)	5’-TATCACAGAGGAGCAACGGG-3’	5’-TCATCAGAGGGAGGAAGAACC-3’
